# One-pot RPA-CRISPR/Cas12a assay for rapid and sensitive detection of *Sclerotium rolfsii* in *Glycine max*

**DOI:** 10.3389/fpls.2026.1927846

**Published:** 2026-09-21

**Authors:** Wenjie Chen, Shufang Chen, Xiayan Qin, Fuyue Tang, Qingyuan Wei, Xiaohong Guo, Rongchang Wei, Yuan Chen, Jiang Liang, Xue Bai

**Affiliations:** Guangxi Academy of Agricultural Science, Nanning, China

**Keywords:** disease early warning, one-pot assay, RPA-CRISPR/Cas12a, *Sclerotium rolfsii*, soybean

## Abstract

Soil-borne fungal diseases are major constraints to the yield of legume crops, and it is therefore urgent to develop rapid and accurate detection methods for plant pathogens. In this study, a one-pot fluorescent detection system combining recombinase polymerase amplification (RPA) with CRISPR/Cas12a was established for the rapid nucleic acid detection of *Sclerotium rolfsii*, the causal agent of soybean southern blight. The results demonstrated that the developed RPA-CRISPR/Cas12a one-pot assay could accurately identify the target pathogen within 45 minutes. The 95% limit of detection (LOD) was estimated to be 126.29 copies per reaction, and no cross-reactivity was observed with other common phytopathogenic fungi. The detection results of field soybean tissue and soil samples showed a consistency rate of 92.42% with the qPCR and full (100 %) agreement with ITS sequencing. Moreover, this assay was capable of detecting latently infected plants that could not be identified by routine field diagnosis, and qualified for pre-planting soil screening. This method is also applicable to the rapid and accurate screening of other phytopathogenic fungi in plant tissues and soil samples, and can be applied to the early warning and prevention of crop diseases.

## 1 Introduction

*Sclerotium rolfsii* (*S. rolfsii*) is a highly pathogenic soil-borne fungus capable of infecting over 500 plant species, which causing sclerotial rot or collar rot or stem rot or southern blight (Punja, 1985; Harlton, 1995). Numerous studies have documented the pathogenicity of *S. rolfsii* in peanut (Le et al., 2012; Veerendrakumar et al., 2025), bean (Garibaldi et al., 2013; Erima et al., 2025), tomato (Sultana and Hossain, 2022; Castello et al., 2025), and other crops such as pepper (Qiu et al., 2024; Jia et al., 2026), *Aconitum carmichaelii* (Wang et al., 2024) and pumpkin (Najera et al., 2018). The pathogen causing southern blight disease on soybean was first reported in 2020 (Zheng et al., 2020). In China, the disease also called “white silk disease”, due to *S. rolfsii* produces white filamentous hyphae that spread radially across infected areas. Under field conditions, soybeans can be infected by *S. rolfsii* throughout their entire growth period, which may lead to plant death. The disease is most severe during the seedling stage, causing broken ridges and rows, ultimately reducing soybean yield. *S. rolfsii* not only has a broad host range and strong pathogenicity but also faces very few immune germplasm resources across different crop species. In crops such as peanuts and soybeans, stable “immune-type” germplasm against *S. rolfsii* has not yet been developed through breeding (Mehan et al., 1995; Patra et al., 2023; Ramteke et al., 2024; Bangaru et al., 2025). In the absence of resistant germplasm, chemical control remains an effective strategy for managing southern blight disease. However, it is essential to quickly determine whether the soil contains *S. rolfsii* before applying seed treatment. At the same time, accurately diagnosing whether dead seedlings observed in soybean production are caused by *S. rolfsii* is a prerequisite for effective chemical control. Therefore, establishing rapid and accurate detection methods for the pathogen and reliable diagnostic techniques for *S. rolfsii* is of great significance for planting planning, disease management, and precise prevention and control. Such advances would provide critical technical support for stabilizing soybean yield and ensuring food and oil safety.

Current conventional diagnostic methods for southern blight of soybean fall into three categories: field symptom diagnosis, pathogen isolation and culture identification, and conventional molecular biological diagnosis. Field symptom diagnosis is the most commonly used method in grassroots agricultural production (Bills et al., 1993). It requires no specialized equipment and is simple to perform. However, the early symptoms of soybean southern blight closely resemble those of other soil-borne diseases such as soybean root rot, Fusarium wilt, and damping-off, making misdiagnosis highly likely. Moreover, field symptom diagnosis cannot detect sclerotia in the soil at an early stage; it can only be performed after plants have already shown symptoms, thus failing to provide early warning. Pathogen isolation and culture identification is the traditional gold standard for laboratory diagnosis, offering high diagnostic accuracy. Nevertheless, it has several drawbacks, including a long culture period (3–7 days) and cumbersome procedures. Furthermore, the isolation rate of sclerotia from soil samples is extremely low, leading to a high risk of false negatives. Conventional molecular biological diagnosis based on PCR technology offers significantly higher sensitivity and specificity than culture-based methods (Aradhika et al., 2021; Tripathi et al., 2023; Wang et al., 2023; Lee and Choi, 2025). However, it requires specialized personnel and equipment, making it difficult for farmers and local agricultural technicians to perform independently. The entire process, from sample collection to result output, typically takes 2–4 hours, which limits its ability to provide timely guidance for field disease management. Moreover, when applied to complex samples such as soil, the accuracy of this method often fails to meet practical application requirements.

The field occurrence characteristics of soybean southern blight of soybean and the limitations of existing diagnostic methods underscore the necessity and significance of developing rapid and accurate diagnostic technologies. The CRISPR-Cas (Clustered Regularly Interspaced Short Palindromic Repeats-CRISPR associated proteins) system, a next-generation molecular diagnostic technology developed in recent years, offers a novel solution for plant disease diagnosis by virtue of its core advantages, including single-base-level target recognition specificity, target-triggered cascade signal amplification, mild reaction conditions, and simple operational procedures (Gootenberg et al., 2017; Chen et al., 2018a; Li et al., 2018; Ansari et al., 2026). The CRISPR-Cas12a system is one of the most widely applied subtypes in the diagnostic field. Guided by crRNA, this system specifically recognizes target double-stranded DNA sequences and activates both the cis-cleavage and trans-cleavage activities of the Cas12a protein, which indiscriminately cleaves single-stranded DNA reporter probes in the system (Chen et al., 2018b). Target detection is achieved through changes in fluorescence or colorimetric signals. Compared with conventional PCR, LAMP, and RPA techniques, CRISPR-Cas12a diagnostics offer irreplaceable advantages: first, exceptionally high specificity with an extremely low false positive rate, enabling accurate discrimination of closely related species and avoiding misdiagnosis; second, extremely high sensitivity that allows precise detection of low-abundance targets, preventing false negatives; and third, the ability to complete reactions at a constant temperature of 37°C without requiring precision thermocyclers (Anbazhagan et al., 2024), meeting the demands of grassroots plant protection agencies and agricultural personnel for simplified detection systems.

To date, CRISPR diagnostic technology has been widely applied and validated in the field of plant disease detection (Tanny et al., 2023; Anbazhagan et al., 2024; Jaybhaye et al., 2024; Kanapiya et al., 2024; Ansari et al., 2026). Researchers have developed CRISPR-based rapid detection systems for a variety of fungal, bacterial, and viral diseases in crops such as rice blast (Karan et al., 2025), wheat stripe rust (Sanchez et al., 2022; Hu et al., 2025), tomato brown rugose fruit virus (Alon et al., 2021; Mehmood et al., 2026), and potato late blight (Chen et al., 2026), demonstrating the feasibility and application value of this technology in plant disease diagnosis. In the field of soybean diseases, CRISPR diagnostic technology has also seen preliminary applications. Researchers have developed CRISPR-based rapid detection systems for diseases such as phytophthora root rot (Guo et al., 2023; Li et al., 2024) and southern stem canker (Sun et al., 2023; Dong et al., 2024), providing technical support for integrated management of soybean diseases. However, CRISPR-based rapid diagnostic systems specifically for *S. rolfsii*, the causal agent of southern blight of soybean, have not been well established. This remains a significant research gap and fails to provide technical support for rapid and accurate field diagnosis of southern blight of soybean.

To the light of above consideration, this study selects the conserved ITS rDNA sequence of *S. rolfsii* as the target and combines the rapid isothermal amplification capability of recombinase polymerase amplification (RPA) with the high specificity and high sensitivity signal amplification ability of Cas12a protein to develop one-pot RPA-Cas12a rapid and accurate diagnostic system for *S. rolfsii*. After validating its specificity, sensitivity and performance with authentic field specimens, we conclude that the one-pot RPA-LbCas12a assay enables reliable on-site identification of *S. rolfsii* from soybean plants and soil. This research not only provides core technical support for rapid integrated field management of southern blight of soybean but also offers a reference for the diagnosis of soil-borne fungal diseases in other crops. It holds significant theoretical and practical value for ensuring the safe production of soybeans and other crops in China and safeguarding national food security.

## 2 Materials and methods

### 2.1 Sample collection

Ten prevalent plant- and soil-borne pathogens, comprising one bacterial strains, two oomycete isolates and seven fungal isolates, were employed for the specificity test. All isolates were authenticated via morphological observation and molecular identification, with full metadata summarized in **Supplementary Table 1**.

For detecting *S. rolfsii* in plant and soil samples, dead soybean seedlings were observed in fields at the Mingyang Baseof the Guangxi Academy of Agricultural Sciences (GAAS) (23°19′00″ N, 107°58′00″ E; altitude 118.32 m; red soil) and at the Mucun Soybean Demonstration Site (Xingye County, Yulin City; 22°45′32″ N, 110°05′08″ E; altitude 110.08 m; red soil), Guangxi Zhuang Autonomous Region, in March 2026. We collected six diseased-asymptomatic sample pairs in Yulin, alongside six and four pairs from separate Nanning field plots. Each pair contained stem-root plant tissues and paired rhizosphere soil. Stem samples: Stem segments spanning 2–3 cm below the cotyledons to 1–2 cm above the root-soil interface were harvested from soybean plants, pooled across biological replicates, and homogenized. Aliquots of 0.1–0.2 g homogenized stem tissue were transferred to pre-filled fungal lysis tubes. Root samples: The entire root system of each plant was aseptically excised using a sterile scalpel, pooled, and homogenized. Then 0.1–0.2 g homogenized root tissue was weighed and loaded into pre-filled fungal lysis tubes. Soil samples: Approximately 0.2 g of soil was weighed and added to soil grinding lysis tubes pre-loaded with ceramic lysis beads.

To further strengthen data reliability, we collected 20 soil samples from two soybean production fields in Lijian Base of GAAS (23°19′00″ N, 107°58′00″ E; altitude 118.32 m; red soil) and a soybean demonstration plot in Longtou Township, Fusui County, Chongzuo City (22°43′03″ N, 107°57′07″ E; altitude 86.07 m; red soil), Guangxi Zhuang Autonomous Region, in July 2026, with 12 samples from Lijian and 8 from Fongtou. No seedling mortality was observed in the Lijian field, whereas extensive seedling death occurred across the Longtou site. For all collections, rhizosphere soil immediately adjacent to soybean roots was sampled; specifically, at the Longtou site, all samples were taken exclusively from the rhizosphere of plants that had succumbed to soilborne root rot diseases.

### 2.2 DNA extraction

The strains were grown at 25°C for 4 days, and the hyphae were scraped with a scalpel into the 2 mL centrifuge tube. For genomic DNA extraction of strains and plant tissues, we used fungal genomic DNA extraction kit (Cat. No. C0130A, TianGen, Beijing, China). Genomic DNA from soil samples was extracted using the BioFast Soil Genomic DNA Extraction Kit (Cat. No. BSC21S1, Bioer Technology, Hangzhou, China). DNA concentration was quantified using NanoDrop™One (Thermo Fisher Scientific, Waltham, MA, USA).

### 2.3 Primer and crRNA design and *in vitro* transcription of crRNA

The ITS region of the ribosomal RNA small subunit of *S. rolfsii* was selected as the candidate target. To improve detection specificity for the target species, BLAST search was performed against the NCBI database, and primer pairs were designed targeting conserved regions specific to *S. rolfsii* while showing variation in other species. Notably, the target amplicon was required to contain at least one protospacer adjacent motif (PAM) site (5′-TTTN-3′) to facilitate LbCas12a recognition (van Dongen et al., 2020).

The crRNA was designed based on the amplification product generated by the candidate primer pair. The reverse complementary DNA sequence of the crRNA and T7 promoter sequence was synthesized to serve as the template strand for *in vitro* transcription of crRNA. *In vitro* transcription and purification of crRNA were performed using the Cas12a High Yield crRNA Synthesis and Purification Kit (Cat. No. 31903, ToloBio, Shanghai, China) according to the manufacturer’s instructions. The complete sequence of the crRNA includes the 5′ direct repeat sequence (scaffold sequence), which is involved in the formation of the Cas12a-crRNA ribonucleoprotein complex, and the 3′ spacer sequence (guide sequence) responsible for target-specific recognition (see **Supplementary Table 1**).

The RPA primers and the template strand for crRNA transcription were synthesized by Sangon Biotech Co., Ltd. (Shanghai, China). The sequences of all primers and crRNAs are listed in **Supplementary Table 2**. A FAM/BHQ1-labeled single-stranded DNA was used as the trans-cleavage probe for RPA-Cas12a-mediated real-time and endpoint fluorescence assays.

### 2.4 Selection of RPA primers for *S. rolfsii*

The RPA assay was performed using the DNA Isothermal Rapid Amplification Kit (Basic) (Cat. No. WLB8201, Amplification Future, Changzhou, China). The reaction mixture, with a total volume of 10 μL, consisted of 5 μL of rehydration buffer (A Buffer), 0.5 μL each of forward and reverse primer (10 μmol/L), 1 μL of template DNA, 2 μL of DEPC-treated water, and 1 μL of B Buffer. The reaction was carried out at 37°C for 30 min in a PCR system (Hongshi, Shanghai, China), and the amplification products were assessed by 2% agarose gel electrophoresis.

### 2.5 Establishment and optimization of the one-pot RPA-LbCas12a assay for *S. rolfsii*

The one-pot RPA-CRISPR/Cas12a assay system comprised 5 μL of rehydration buffer (A Buffer), 0.5 μL each of forward and reverse primer (10 μmol/L), 1 μL of template DNA, 1 μL of LbCas12a (1 μM) (32108, ToloBio, Shanghai, China), 0.5 μL of crRNA (10 μM), 0.5 μL of ssDNA probe (10 μM, 5 ‘FAM- CCCCCCCC -3’BHQ1), and 1 μL of B Buffer. The reaction was performed at 37°C for 45 min, and fluorescence signals in the FAM channel were monitored every 30 s using a SLAN-96P Real-Time PCR System (Shanghai Hongshi Medical Technology Co., Ltd., Shanghai, China). To optimize the reaction conditions, ten different crRNAs were tested, and the serial concentrations of LbCas12a were optimized (0.05-1.5 μM), with final working concentrations set at 150, 100, 50, 30, 10 and 5 nM. The fluorescence signal intensity under each condition was subsequently evaluated.

### 2.6 Specificity and sensitivity of the one-pot RPA-LbCas12a assay for *S. rolfsii*

For specificity assessment, genomic DNA from ten common pathogens, and two plasmids containing the ITS region of the small subunit ribosomal RNA gene from *Sclerotium coffeicola* (*S.coffeicola*) (strain BOS-AYA001, GenBank: LC835020.1) and *Sclerotium delphinii* (*S. delphinii*) (stain SC1, GenBank: KP176676.1)—the closest phylogenetic relatives of *S. rolfsii*, were tested using the established one-pot RPA-LbCas12a assay. Three independent replicate reactions were performed for each pathogen. The recombinant plasmid containing the target sequence served as the positive control (PC), and deionized water was used as the no-template control (NTC).

For sensitivity evaluation, the recombinant plasmid containing the target sequence of the *S. rolfsii* ITS gene was serially diluted to concentrations of 5, 00, 250, 200, 100, 50, and 25 copies/μL. Each dilution was tested using the aforementioned assay system to determine the analytical sensitivity. Logistic regression analysis was performed using MedCalc (version 16.4) to model the relationship between detection probability and plasmid concentration. The limit of detection (LOD) was defined as the concentration at which the probability of a positive result reached 95%. Four to eight replicate reactions were carried out for each concentration, with sterile water serving as the negative control. To comparatively evaluate the sensitivity, the same serially diluted recombinant plasmids were simultaneously tested using a qPCR assay targeting the ITS region of *S. rolfsii* (Luo et al., 2022). The qPCR reactions were performed in a 20 μL volume containing 10 μL of 2× Magic Green Taq SuperMix (Cat. No. 21052, ToloBio, Shanghai, China), 0.5 μL of 10 μM forward primer, 0.5 μL of 10 μM reverse primer, 0.2 μL of SybrGreen I (Cat. No. A616690-1000, Sangon Biotech, Shanghai, China), and 2 μL of template DNA. Cycling conditions were set as follows: initial denaturation at 95°C for 3 min; followed by 45 cycles of 95°C for 15 s, 55°C for 15 s, and 72°C for 30 s; with a final extension at 72°C for 5 min. Each concentration was run in triplicate, and sterile water served as the negative control. The Cycle threshold (Ct) values were recorded, and the limit of detection (LOD) for qPCR was determined as the concentration corresponding to a 95% positivity rate via logistic regression.

### 2.7 Amplicon sequencing of the plant tissues and soil DNA samples

Soybean tissue and soil samples were subjected to amplicon sequencing. The fungal Internal Transcribed Spacer (ITS) region was amplified using primers ITS1F (5′-CTTGGTCATTTAGAGGAAGTAA-3′) and ITS2R (5′-GCTGCGTTCTTCATCGATGC-3′). The PCR cycling conditions were as follows: initial denaturation at 95°C for 10 min; followed by 28 cycles of 95°C for 15 s, 55°C for 10 s, and 72°C for 10 s; with a final extension at 72°C for 10 min. Following purification and quantification using the Qubit system, sequencing libraries were constructed using the NE-NET Flex™ Rapid DNA-Seq Kit and sequenced on an Illumina MiSeq PE300 platform (Majorbio, Shanghai, China). Raw demultiplexed data were subjected to quality control using fastp (Chen et al., 2018). Paired-end reads were then merged using FLASH (Magoc and Salzberg, 2011). Subsequently, denoising was performed using the DADA2 plugin (Dillon et al., 2019) within Qiime2 to obtain Amplicon Sequence Variants (ASVs). Chloroplast and mitochondrial sequences were filtered out. Taxonomic classification of ASVs was performed using a Naïve Bayes classifier trained on the SILVA v138 and UNITE databases. All bioinformatic analyses were conducted on the Majorbio Cloud Platform (https://v.majorbio.com/user-center/login?redirect_url=https://v.majorbio.com/project-center/overview). Finally, species abundance tables were downloaded for quantitative analysis of *S. rolfsii* abundance.

### 2.8 Application of the one-pot RPA-LbCas12a assay for detecting *S. rolfsii* in plant and soil samples

DNA extracted from samples collected at the Mingyang Base, Lijian Base, Mucun Base, and the Longtou Township demonstration plot was tested using the one-pot RPA-LbCas12a assay. For comparison, the same samples were also validated by qPCR using primers (SCR-F: CGTAGGTGAACCTGCGGA; SCR-R: CATACAAGCTAGAATCCC) reported in the literature (Luo et al., 2022).

## 3 Results

### 3.1 The principle and workflow of one-pot RPA-LbCas12a assay for *S. rolfsii*

The principle and workflow of the integrated RPA and CRISPR/Cas12a system for detecting *S. rolfsii* are illustrated in **Figure 1**. Briefly, genomic DNA of *S. rolfsii* was extracted from soybean stem and root tissues, as well as their corresponding soil samples, using commercial fungal or soil DNA extraction kits. The detection platform, which combines RPA with CRISPR/LbCas12a-based detection, allows for one-pot, one-step nucleic acid detection at a constant temperature. The RPA-amplified products are specifically recognized by the corresponding LbCas12a/crRNA system, thereby activating the Cas12a protein and triggering trans-cleavage of the reporter DNA. The reporter DNA, labeled with a fluorophore (6-FAM) and a quencher (BHQ1), emits fluorescence upon cleavage. The fluorescence signals can be detected using a real-time PCR fluorescence reader or other compact, portable blue-light instruments. The entire process, including rapid template preparation (15 min), RPA-CRISPR/Cas12a reaction, and result detection (45 min), can be completed within 1 hour.

**Figure 1:**
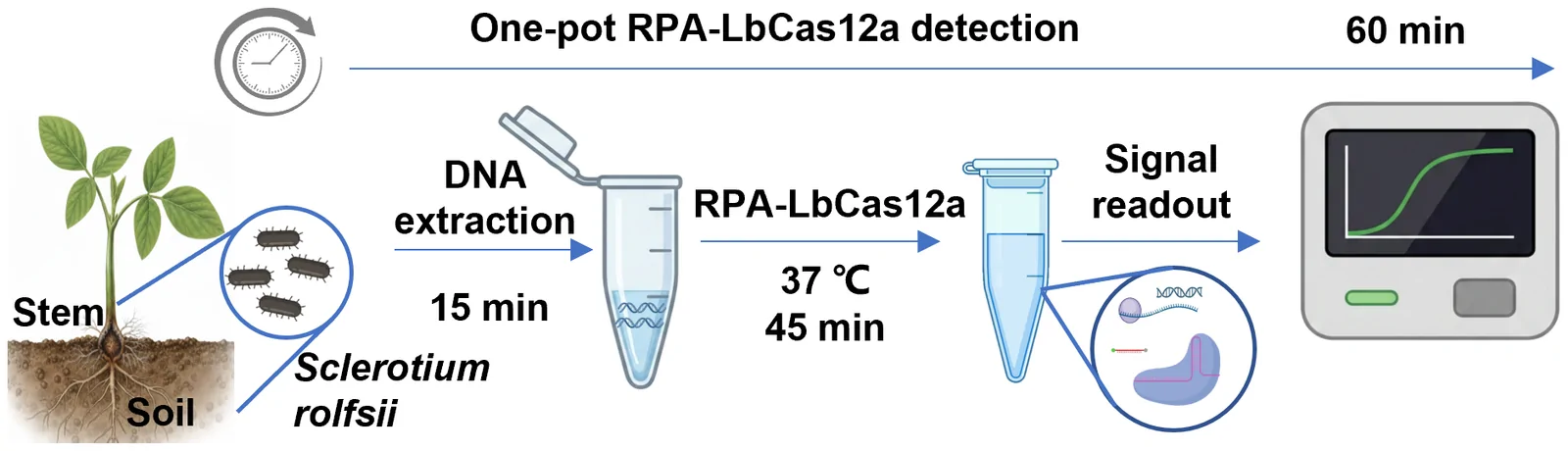
Schematic workflow of the one-pot RPA-CRISPR/LbCas12a detection platform. Genomic DNA was extracted from infected soybean stem tissues and soil samples with a fungal genomic DNA extraction kit. The integrated one-pot RPA-LbCas12a reaction was incubated at 37°C for 45 min to realize synchronous target amplification and cleavage-mediated signal readout via real-time fluorescence measurement. The whole sample-to-result detection workflow can be finished within 1 h.

### 3.2 Establishment and optimization of a one-pot RPA-LbCas12a assay for the detection of *S. rolfsii*

Based on the conserved region of the *S. rolfsii* ITS gene, four forward (F1–F4) and four reverse (R1–R4) primers were designed for the RPA assay. These primers were combined pairwise to form sixteen primer combinations, which were then evaluated for ITS amplification. To identify the optimal combination, the amplification products were assessed by agarose gel electrophoresis. Based on product yield and specificity, the F3/R4 combination was selected as the best-performing primer set (**Figure 2A**), exhibiting both high amplification efficiency (strong band intensity) and high specificity (absence of non-specific amplification bands).

**Figure 2:**
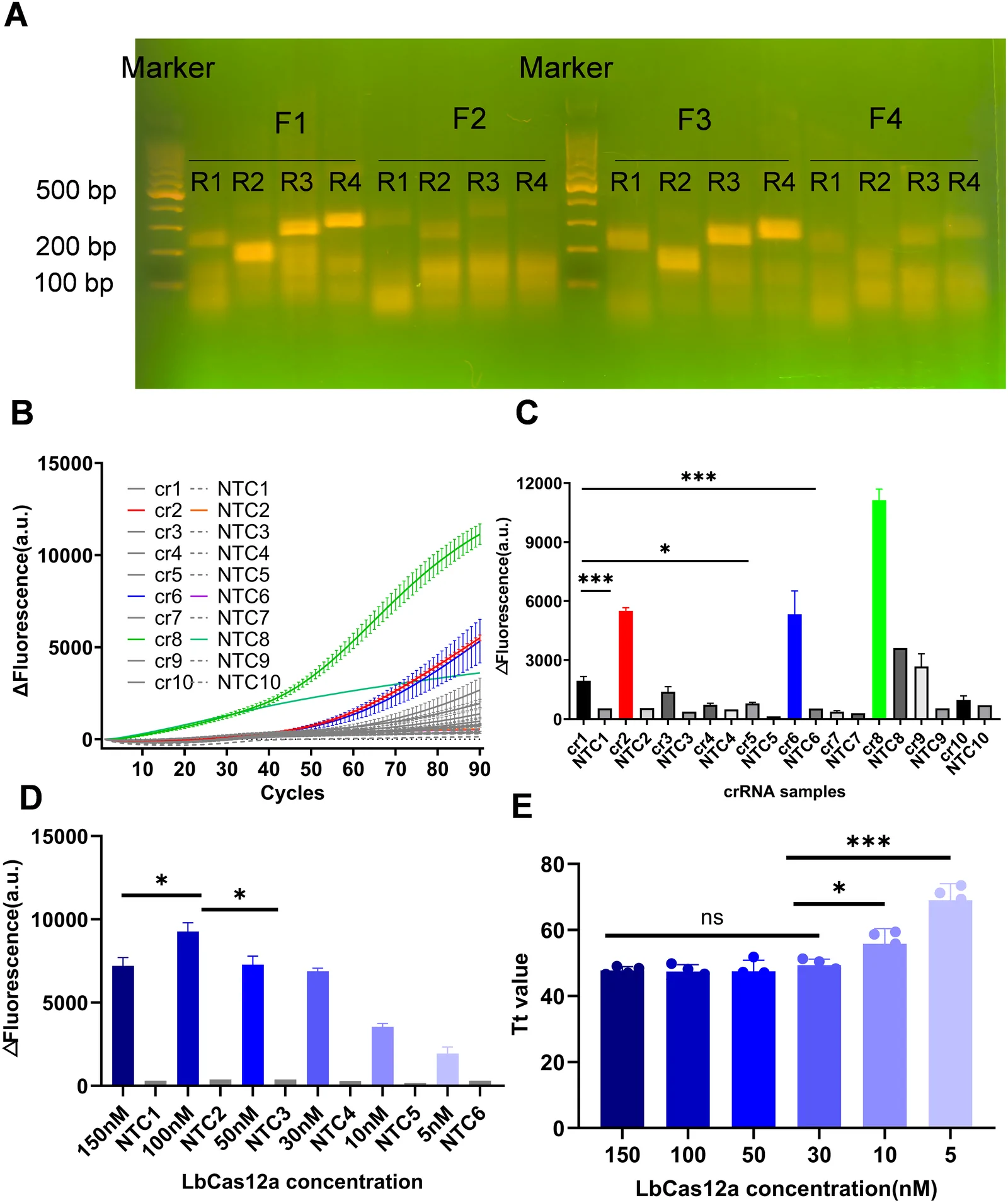
Establishment and optimization of the one-pot RPA-CRISPR/LbCas12a detection system targeting *S. rolfsii.* **(A)** Agarose gel electrophoresis analysis of amplicons generated by distinct RPA primer pairs. **(B)** Real-time fluorescence curves and **(C)** endpoint fluorescence shift values for crRNA screening in the one-pot RPA-Cas12a assay. **(D, E)** Optimization of LbCas12a dosage for the integrated one-pot reaction. **(D)** Endpoint fluorescence shift value across a gradient of LbCas12a concentrations. **(E)** Time-to-positivity (Tt) values corresponding to different LbCas12a concentrations.All data are shown as mean ± s.d. (n ≥ 3). NTC, no-template control. Statistical significance: *P < 0.05, **P < 0.01, ***P < 0.001, ****P < 0.0001.

Derived from the ITS amplicon sequence, ten Cas12a crRNAs (crRNA1–crRNA10) were designed for evaluation. When tested in the one-pot RPA-Cas12a detection system, crRNA2, crRNA6, and crRNA8 generated the strongest positive signals among all crRNAs (**Figure 2B**). However, crRNA8 exhibited high background signal in the no-template control (NTC), and crRNA6 showed poor repeatability. Therefore, crRNA2 was selected for use in the one-pot detection system (**Figure 2C**).

To determine the optimal the concentrations of Cas12a protein, the Cas12a protein were diluted into four different concentrations, including 150 nM, 100 nM, 50 nM, 30 nM and 10 nM in the reaction. As shown in **Figures 2D, E**, the optimum reaction concentrations of Cas12a protein in the dose of 100 nM, as it produced the highest fluorescence intensity and lower Tt value (shorter time to positivity).

### 3.3 Specificity and sensitivity analysis of the one-pot RPA-LbCas12a assay for *S. rolfsii* detection

To evaluate the specificity of the one-pot RPA-LbCas12a system for *S. rolfsii*, ten common fungal pathogens were collected as interfering nucleic acid samples, including *C. jasminigenum*, *C. jasminigenum*, *D. longicolla*, *F. oxysporum*, *F. solani*, *P. sojae*, *P. ultimum*, *R. microsporus*, *P. syringae*, *P. pachyrhizi*. A recombinant plasmid containing the target sequence of *S. rolfsii* served as the positive control (PC), and nuclease-free water served as the NTC. As illustrated in **Figure 3A**, only the PC samples produced a significant fluorescence intensity, while no significant signal was observed in the interfering samples. These results demonstrated that the one-pot assay was highly specific for *S. rolfsii* detection, without any cross-reactivity with other nucleic acid samples.

**Figure 3:**
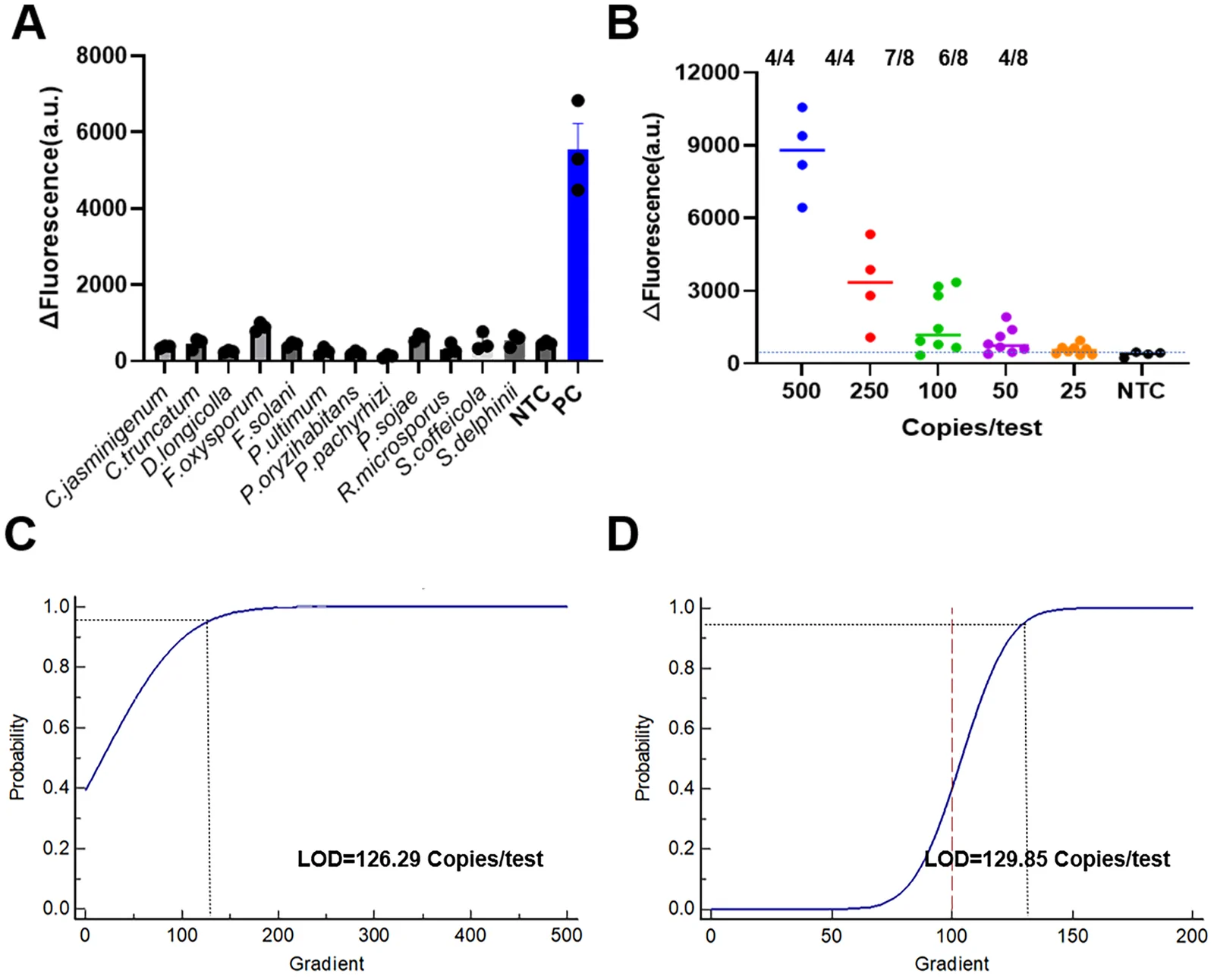
Specificity and sensitivity evaluation of the one-pot RPA-LbCas12a assay targeting *S. rolfsii*. **(A)** Cross-reactivity test of the one-pot RPA-LbCas12a assay against diverse fungal and bacterial pathogens. **(B)** Detection signals and corresponding positive detection rates (N1/N2: number of positive replicates/total tested replicates) of the one-pot RPA-LbCas12a system with serially diluted target templates. Limit of detection (LOD) determination of *S. rolfsii* using the one-pot RPA-LbCas12a assay **(C)** and qPCR **(D)**.

To determine the sensitivity of the one-pot RPA-LbCas12a assay for *S. rolfsii*, the recombinant plasmid was serially diluted to 500, 250, 100, 50, and 25 copies per test, respectively. Four to eight replicates were performed at each dilution level. In the one-pot RPA-Cas12a assay, 100% positive results were obtained at 500 and 250 copies per test, while 7 out of 8 positive results were observed at 100 copies per test and 6 out of 8 at 50 copies per test (**Figure 3B**). Subsequently, sigmoid plot analysis determined the LOD to be 126.29 copies per test at a 95% probability level (**Figure 3C**). For methodological comparison, we conducted parallel LOD quantification via qPCR, with the results presented in **Figure 3D**. The qPCR assay achieved an LOD of 129.85 copies per test, comparable to that of our one-pot RPA-LbCas12a system.

### 3.4 Validation of the *S. rolfsii* one-pot RPA-LbCas12a assay for plant and soil samples

Southern blight of soybean caused by *S. rolfsii* poses severe agricultural threats across the Guangxi Zhuang Autonomous Region of China. In this study, 22 paired sample sets consisting of soybean stems, roots, and matched rhizosphere soil were collected from two soybean fields in Nanning and Yulin Cities. Twelve of these sample sets were collected from symptomatic soybean plants, while the remaining ten were from asymptomatic individuals. Genomic DNA extracted from each sample was subjected to testing to validate the diagnostic capability of our one-pot RPA-LbCas12a CRISPR assay for identifying *S. rolfsii* in plant tissues and rhizosphere soil. Conventional qPCR was performed in parallel on all samples as a reference comparison (**Supplementary Table 3**).

ROC curve analyses revealed that the one-pot RPA-LbCas12a assay achieved a diagnostic sensitivity of 94.44% (95% CI: 81.3–99.3), outperforming qPCR, which yielded a sensitivity of only 86.11% (95% CI: 70.5–95.3) (**Figures 4A, B**). For all symptomatic samples collected from both sampling sites, the CRISPR-based assay delivered a 94.44% (34/36) positive detection rate. In contrast, qPCR failed to identify five infected samples, comprising one rhizosphere soil sample and two root tissue specimens (**Figure 4C**). Notably, the CRISPR-based assay returned positive signals in both root and stem tissues of one asymptomatic sample set (YH5), implying latent early-stage infection before visible disease symptoms emerge. Collectively, the detection results of field soybean tissue and soil samples showed a consistency rate of 92.42% with the qPCR method. Notably, samples yielding inconsistent outcomes between the two detection assays were further validated via ITS sequencing. Sequencing-derived identification exhibited 100 % concordance with the CRISPR-based detection results (**Figure 4C**). These results demonstrate that the one-pot RPA-LbCas12a assay represents a robust and practical diagnostic tool for on-site identification of *S. rolfsii* in field-grown soybean and soil.

**Figure 4:**
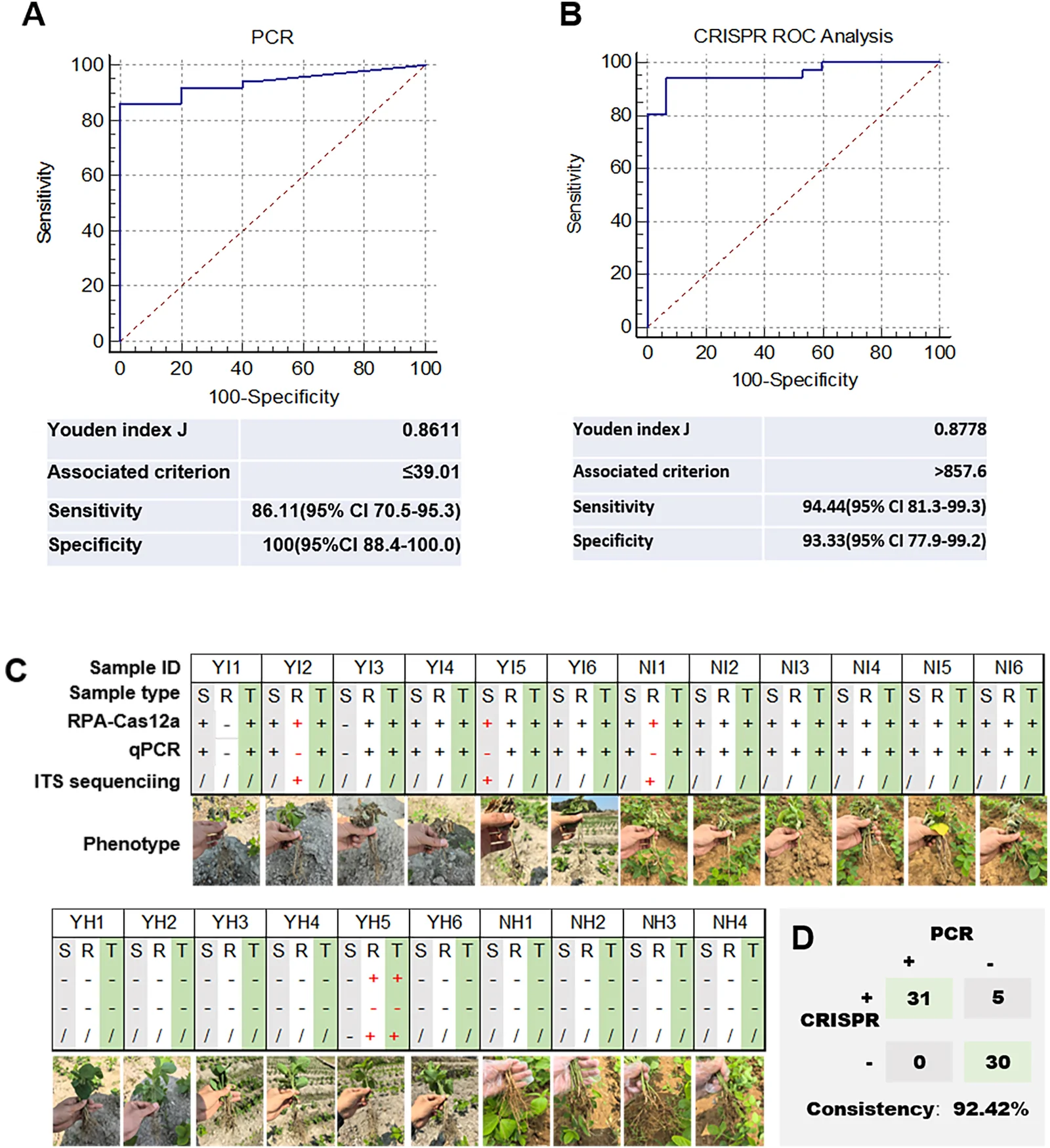
ROC curve analysis comparing the diagnostic performance of qPCR **(A)** and one-pot RPA-LbCas12a **(B)** for *S. rolfsii* samples. **(C)** Nucleic acid detection results obtained via qPCR and one-pot RPA-LbCas12a (CRISPR), alongside the phenotypic status of healthy and *S. rolfsii*-infected soybean plants and corresponding rhizosphere soil samples. Label definitions: 0 = negative, 1 = positive. All specimens were collected from Nanning and Yulin, Guangxi Zhuang Autonomous Region. Sample cohorts included six infected groups (NI1–NI6) and four healthy groups (NH1–NH4) from Nanning, as well as six infected groups (YI1–YI6) and six healthy groups (YH1–YH6) from Yulin. Each group contained three types of samples: soil (gray), root tissue (white), and stem tissue (green). **(D)** Diagnostic concordance between CRISPR-based detection and conventional quantitative PCR.

Building upon the above findings, we extended our sample panel by collecting 20 soil specimens from experimental plots located in Lijian Base of GAAS (Wuming District, Nanning) and Longtou Township (Fusui County, Chongzuo), Guangxi Zhuang Autonomous Region. All specimens were subjected to parallel testing using our one-pot RPA-Cas12a assay and ITS sequencing, and raw data are summarized in **Supplementary Table 4**. The RPA-CRISPR/Cas12a assay showed excellent concordance with conventional PCR and NGS results. This outcome proves our CRISPR-based rapid assay is qualified for pre-planting soil screening. Early-stage pathogen surveillance facilitates the preemptive management of soil-borne fungal pathogens and carries substantial practical value for field-scale disease prevention.

## 4 Discussion

Leguminous crops including soybean serve as crucial sources of dietary protein, edible oil and feed protein for livestock worldwide (Hartman et al., 2011). Fungal diseases not only cause substantial yield losses of legumes, but also pose severe threats to food and feed safety for humans and animals. Among various fungal diseases, sclerotium rot, basal stem rot, stem rot and southern blight of soybean caused by *S. rolfsii* are particularly destructive. This pathogen is widely distributed across temperate, tropical and subtropical regions globally (Patra et al., 2023), and has become a major biotic stress limiting legume production around the world. Given the enormous challenges in breeding for fungal disease resistance, timely diagnosis combined with chemical control remains an effective strategy to mitigate disease losses in agricultural practice.

Sclerotia residing in soil serve as the primary inoculum of southern blight of soybean, whereas contaminated seeds and latently infected seedlings act as critical vectors for pathogen dissemination. Conventional field diagnostic methods fail to accurately detect these primary infection sources. Therefore, rapid and precise detection technologies targeting soil, seeds and seedlings are urgently needed to forecast disease risks in advance and achieve source-based disease management. CRISPR-based nucleic acid detection has emerged as a powerful molecular tool owing to its outstanding specificity, high sensitivity and convenient operation (Fu et al., 2026). Herein, we established a novel RPA-CRISPR/Cas12a assay for the rapid detection of *S. rolfsii*. This system enables highly sensitive and specific detection in plant and soil samples, and the entire procedure from nucleic acid extraction to target detection can be completed within 1 hour, which fully meets the requirements for rapid and accurate pathogen detection.

CRISPR-based molecular detection has been widely applied in the identification of crop pathogens, allelic genotyping and genetically modified component testing, including numerous studies on CRISPR assays for fungal pathogens (Tanny et al., 2023; Ansari et al., 2026). Nevertheless, CRISPR diagnostic methods specifically for *S. rolfsii* have not been reported to date, while relevant CRISPR nucleic acid detection techniques for other soybean fungal pathogens are available. In 2023, Guo et al. first developed an RPA-CRISPR/Cas12a assay for Phytophthora soja (Guo et al., 2023). This two-tube two-step system performs RPA amplification and CRISPR detection in separate tubes, which is cumbersome to operate and prone to aerosol contamination. In the following year, Li et al. placed RPA reagents at the tube bottom and CRISPR components on the inner tube cap (Li et al., 2024). After RPA amplification, centrifugation mixes the two reaction systems to initiate CRISPR detection, establishing a one-tube two-step assay for *P. sojae*. This approach effectively avoids aerosol contamination caused by repeated tube opening and pipetting. Similarly, several research groups also constructed two-tube two-step or one-tube two-step RPA-CRISPR/Cas12a assays for *Diaporthe aspalathi* and *Diaporthe caulivora*, the causal agents of soybean southern stem rot (Sun et al., 2023; Dong et al., 2024).

Although the one-tube two-step format eliminates repeated pipetting and aerosol contamination risks, the physical separation of RPA and CRISPR components as well as the sequential reaction procedures complicate the detection workflow, and bring considerable challenges to production and commercialization of detection kits. Due to the inherent incompatibility between RPA amplification and CRISPR cleavage systems, the development of one-pot integrated detection assays has long been a key research focus and technical bottleneck in CRISPR diagnostics (Huang et al., 2025a; Liu et al., 2025; Chen et al., 2026).

In this study, we screened optimal primer pairs with high amplification efficiency and candidate crRNAs that support one-pot detection. The incompatibility between RPA and CRISPR systems mainly arises from the cis-cleavage activity of CRISPR/Cas, which degrades target templates, impairs amplification efficiency and ultimately reduces assay sensitivity. Generally, one-tube two-step assays exhibit higher sensitivity than one-pot systems. To improve the performance of one-pot detection, multiple innovative strategies have been proposed recently, such as selection of suboptimal PAM (Lu et al., 2022), Cas protein engineering (Cao et al., 2025), crRNA modification (Ma et al., 2025), as well as light and temperature regulation (Hu et al., 2022; Huang et al., 2025b). These strategies are proven effective and contribute to elucidating the regulatory mechanisms of CRISPR detection, and their core principle is to suppress the cis-cleavage activity of CRISPR/Cas.

Our optimization experiments revealed that excessively high concentrations of Cas12 protein compromised the performance of the one-pot assay, which was probably attributed to enhanced cis-cleavage activity that inhibited nucleic acid amplification. By contrast, insufficient Cas12 protein also led to poor detection performance, because low trans-cleavage activity weakened signal amplification. Accordingly, we optimized Cas12a protein concentration and significantly improved the sensitivity of the one-pot system. Our results demonstrated that excessive Cas cis-cleavage activity at 150 nM significantly suppressed RPA amplification, whereas 100 nM Cas achieved an optimal balance between RPA amplification and CRISPR cleavage. The successful establishment of the one-pot system also relied on rigorous screening of primers and crRNAs. High-efficiency primers rapidly enriched target templates and thereby mitigated the adverse effects of cis-cleavage. Meanwhile, crRNA performance directly determined the final detection signal quality. Although crRNA8 yielded strong positive signals, it exhibited elevated non-specific background signals in the no-template control, leading to a low signal-to-noise ratio, which was likely attributed to insufficient specificity. While crRNA6 also produced robust positive signals, it showed poor repeatability, possibly due to unstable secondary structures. Collectively, primer screening, crRNA selection, and Cas concentration optimization represent simpler and more feasible strategies for one-pot CRISPR diagnostic development than complicated engineering or spatiotemporal regulation approaches. The detection results of field samples were highly consistent with those obtained by qPCR and field disease symptoms, verifying the reliability and applicability of this assay for field diagnosis.

## 5 Conclusions

In conclusion, this study established an RPA-CRISPR/Cas12a-based rapid nucleic acid detection assay for *S. rolfsii*, the pathogen of southern blight of soybean. The practical potential of this technique for early and precise pathogen diagnosis was validated using field plant and soil samples. Moreover, this assay provides a valuable reference for pathogen detection in other crops.

Field-deployable point-of-care testing (POCT) is the ultimate goal of CRISPR-based rapid nucleic acid detection. It requires simplified sample pretreatment and visual readout of detection results. In the present work, nucleic acids were extracted using commercial kits and signal readout was performed via a quantitative PCR instrument. In future research, we will optimize nucleic acid release reagents to simplify sample processing, integrate lateral flow strips, portable temperature control and fluorescence readers, and combine smartphone and artificial intelligence technologies to realize intelligent all-in-one detection. This will facilitate on-site, rapid and accurate diagnosis of crop pathogens in field conditions.

## Data Availability

The original contributions presented in the study are included in the article/**Supplementary Material**. Further inquiries can be directed to the corresponding authors.
