# Supplementary material for: One-pot RPA-CRISPR/Cas12a assay for rapid and sensitive detection of Sclerotium rolfsii in Glycine max
Source: Front Plant Sci. 2026 Sep 21;17:1927846. doi: 10.3389/fpls.2026.1927846 (PMC13635342; doi:10.3389/fpls.2026.1927846)
Supplement: Supplementary file 1 [file Table1.docx]

­Electronic Supporting Information for

Rapid and accurate diagnosis of the soil-borne fungus Sclerotium rolfsii in crops using a one-pot RPA-CRISPR/Cas12a assay: a case study on soybean

Table S1. Non-target microorganisms used to validate the one-pot RPA-CRISPR/Cas12a assay

| Species | Abbreviation | Taxonomy | Strain ID | GenBank Acc. No. | Source/Origin |
| --- | --- | --- | --- | --- | --- |
| Fusarium solani (Mart.) Appel & Wollenw | F. solani | Fungi | GF7-11 | PV616807.1 | Kindly provided by Prof. Ye Wenwu's laboratory, Nanjing Agricultural University |
| Phytophthora sojae Kaufmann & Gerdemann | P. sojae | Oomycota | P6497 | KU211331.1 | Kindly provided by Prof. Ye Wenwu's laboratory, Nanjing Agricultural University |
| Pythium ultimum | P. ultimum | Oomycota | F18-6 (Feng et al., 2020) | — | Kindly provided by Prof. Ye Wenwu's laboratory, Nanjing Agricultural University |
| Fusarium oxysporum Schltdl | F. oxysporum Schltdl | Fungi | SMFO9 | MT371379.1 | Kindly provided by Prof. Ye Wenwu's laboratory, Nanjing Agricultural University |
| Colletotrichum truncatum (Schwein.) Andrus & W.D. Moore | C. truncatum | Fungi | 2597AH-1 | PZ886553 | kindly provided by the laboratory of Professor Wang Wubin at Nanjing Agricultural University |
| Colletotrichum plurivorum | C. plurivorum | Fungi | MYC05-3 | PZ899137 | Independently collected from Mingyang, Jiangnan District, Nanning City, Guangxi |
| Diaporthe sojae | D. sojae | Fungi | MYD18-2 | PZ899138 | Independently collected from Mingyang, Jiangnan District, Nanning City, Guangxi |
| Pseudomonas oryzihabitans | P. oryzihabitans | Bacteria | MYP-C6 | PZ785111.1 | Independently collected from Mingyang, Jiangnan District, Nanning City, Guangxi |
| Microsphaera diffusa Cooke&Peck | M. diffusa | Fungi | — | — | Independently collected from Maijiu Town, Xingye County, Guangxi |
| Rhizopus microsporus | R. microsporus | Fungi | Y1 | PV948947.1 | Isolated from Huangyao Town, Zhaoping County, Guangxi |

Table S2. Sequences of primers and crRNAs used in this work.

| Primer ID | Sequence (5'-3') |
| --- | --- |
| Rolfsii-RPA-F1 | CGATAAGTAATGTGAATTGCAGAATCCAGTGA |
| Rolfsii-RPA-F2 | AATATACAACTTTCAACAACGGATCTCTTGGC |
| Rolfsii-RPA-F3 | AATTGCAGAATCCAGTGAATCATCGAATCTTT |
| Rolfsii-RPA-F4 | AATGCGATAAGTAATGTGAATTGCAGAATCCA |
| Rolfsii-RPA-R1 | CCGTAGGCATTTCTACATGTCCTACTAATAGT |
| Rolfsii-RPA-R2 | AGAATATACTCTAACCAGCAACTCTCACATCC |
| Rolfsii-RPA-R3 | CTTCCGGTATAGGCGTAGACATATTATCACAC |
| Rolfsii-RPA-R4 | TAAGTAGTACATACAAGCTAGAATCCCCTTCC |
| Rolfsii-crRNA1 | UAAUUUCUACUAAGUGUAGAU**CGCCCUUUGGUAUUCCGAGG** |
| Rolfsii-crRNA2 | UAAUUUCUACUAAGUGUAGAU**CGAGGGGCAUGCCUGUUUGA** |
| Rolfsii-crRNA3 | UAAUUUCUACUAAGUGUAGAU**AAUUCUCAACCUUACAAAUU** |
| Rolfsii-crRNA4 | UAAUUUCUACUAAGUGUAGAU**UCAACCUUACAAAUUUUUGU** |
| Rolfsii-crRNA5 | UAAUUUCUACUAAGUGUAGAU**CAAAUUUUUGUAUUUGUCAA** |
| Rolfsii-crRNA6 | UAAUUUCUACUAAGUGUAGAU**GAGUAUAUUCUGACUGGCUC** |
| Rolfsii-crRNA7 | UAAUUUCUACUAAGUGUAGAU**GUAGGACAUGUAGAAAUGCC** |
| Rolfsii-crRNA8 | UAAUUUCUACUAAGUGUAGAU**UCACACCAACCGUAGGCAUU** |
| Rolfsii-crRNA9 | UAAUUUCUACUAAGUGUAGAU**ACAAAUACAAAAAUUUGUAA** |
| Rolfsii-crRNA10 | UAAUUUCUACUAAGUGUAGAU**AGAAUUUAAUGACUCUCAAA** |
| Rolfsii-PCR-F1 | CATCGATGAAGAACGCAGCG |
| Rolfsii-PCR-R1 | AGCTAGAATCCCCTTCCGGT |
| Rolfsii-PCR-F2 | CGTAGGTGAACCTGCGGA |
| Rolfsii-PCR-R2 | CATACAAGCTAGAATCCC |

* The underlined sequence is the spacer sequence.

Table S3. Nucleic acid detection results obtained via qPCR and one-pot RPA-LbCas12a (CRISPR)

| Sample ID | Sample type | CRISPR ΔRn | CRISPR result | PCR Ct | PCR result |
| --- | --- | --- | --- | --- | --- |
| NH1 | Soil | 719.9 | 0 | 40.99 | 0 |
|  | Root | 234.3 | 0 | 42.68 | 0 |
|  | Stem | 71.9 | 0 | 43.23 | 0 |
| NH2 | Soil | 727.4 | 0 | 40.58 | 0 |
|  | Root | 193.3 | 0 | 40.75 | 0 |
|  | Stem | -44.5 | 0 | 42.85 | 0 |
| NH3 | Soil | 461.5 | 0 | 45 | 0 |
|  | Root | 508.4 | 0 | 43.66 | 0 |
|  | Stem | 60.7 | 0 | NoCt | 0 |
| NH4 | Soil | 336.7 | 0 | 43.33 | 0 |
|  | Root | 269.4 | 0 | 43.26 | 0 |
|  | Stem | -4.6 | 0 | NoCt | 0 |
| NI1 | Soil | 8244.6 | 1 | 29.5 | 1 |
|  | Root | 1192 | 1 | 42.41 | 0 |
|  | Stem | 14056.5 | 1 | 24.18 | 1 |
| NI2 | Soil | 7761 | 1 | 27.75 | 1 |
|  | Root | 14538.5 | 1 | 30.58 | 1 |
|  | Stem | 13449.6 | 1 | 23.04 | 1 |
| NI3 | Soil | 7920.1 | 1 | 25.7 | 1 |
|  | Root | 11999.6 | 1 | 31.5 | 1 |
|  | Stem | 12459.5 | 1 | 19.38 | 1 |
| NI4 | Soil | 8049.5 | 1 | 30.62 | 1 |
|  | Root | 10401 | 1 | 25.56 | 1 |
|  | Stem | 12436.3 | 1 | 26.26 | 1 |
| NI5 | Soil | 8169.7 | 1 | 29.16 | 1 |
|  | Root | 12060.8 | 1 | 29.29 | 1 |
|  | Stem | 14103.7 | 1 | 26.34 | 1 |
| NI6 | Soil | 4407.4 | 1 | 30.08 | 1 |
|  | Root | 13675.6 | 1 | 37.26 | 1 |
|  | Stem | 12634.2 | 1 | 29.86 | 1 |
| YH1 | Soil | 369.7 | 0 | 45 | 0 |
|  | Root | 434.5 | 0 | NoCt | 0 |
|  | Stem | 513.6 | 0 | NoCt | 0 |
| YH2 | Soil | 594.9 | 0 | 45 | 0 |
|  | Root | 526.7 | 0 | NoCt | 0 |
|  | Stem | 136.7 | 0 | NoCt | 0 |
| YH3 | Soil | 496.1 | 0 | 45 | 0 |
|  | Root | 547.8 | 0 | NoCt | 0 |
|  | Stem | 329.6 | 0 | NoCt | 0 |
| YH4 | Soil | 562 | 0 | 45 | 0 |
|  | Root | 648.2 | 0 | 44.04 | 0 |
|  | Stem | 857.6 | 0 | NoCt | 0 |
| YH5 | Soil | 511.8 | 0 | 43.09 | 0 |
|  | Root | 1804.4 | 1 | 41.16 | 0 |
|  | Stem | 1193 | 1 | 41.04 | 0 |
| YH6 | Soil | 484.1 | 0 | 41.67 | 0 |
|  | Root | 565.3 | 0 | NoCt | 0 |
|  | Stem | 282 | 0 | NoCt | 0 |
| YI1 | Soil | 1046.2 | 1 | 32.3 | 1 |
|  | Root | 473.9 | 0 | NoCt | 0 |
|  | Stem | 7555.2 | 1 | 23.84 | 1 |
| YI2 | Soil | 1062.6 | 1 | 34.72 | 1 |
|  | Root | 1088.1 | 1 | NoCt | 0 |
|  | Stem | 9125 | 1 | 21.52 | 1 |
| YI3 | Soil | 430.3 | 0 | 43.36 | 0 |
|  | Root | 9079.5 | 1 | 33.51 | 1 |
|  | Stem | 7532.5 | 1 | 27.81 | 1 |
| YI4 | Soil | 2280.7 | 1 | 34.88 | 1 |
|  | Root | 21075.9 | 1 | 24.67 | 1 |
|  | Stem | 8817.1 | 1 | 20.06 | 1 |
| YI5 | Soil | 1030.7 | 1 | 41.93 | 0 |
|  | Root | 5804.1 | 1 | 36.43 | 1 |
|  | Stem | 10617.7 | 1 | 28.12 | 1 |
| YI6 | Soil | 1820.2 | 1 | 39.01 | 1 |
|  | Root | 14118.7 | 1 | 33.43 | 1 |
|  | Stem | 12459.7 | 1 | 21.7 | 1 |

ΔRn represents the difference in fluorescence intensity measured at the endpoint relative to the starting baseline, with units of arbitrary units (a.u.). Label definitions: 0 = negative, 1 = positive. All specimens were collected from Nanning and Yulin, Guangxi Zhuang Autonomous Region. Sample cohorts included six infected groups (NI1–NI6) and four healthy groups (NH1–NH4) from Nanning, as well as six infected groups (YI1–YI6) and six healthy groups (YH1–YH6) from Yulin. Each group contained three types of samples.

Table S4. Comparative analysis of the RPA-CRISPR/Cas12a assay, conventional PCR, and NGS for the detection of S. rolfsii in soil samples.

| Sample ID | Collection Site | CRISPR ΔRn | CRISPR Result（+/-） | Conventional PCR Ct | Conventional PCR Result（+/-） | Total Reads | S. rolfsii reads | Relative Abundance (%) | NGS Confimation (+/-) |
| --- | --- | --- | --- | --- | --- | --- | --- | --- | --- |
| S1 | Lijian Base of GAAS | 386.73 | - | NoCt | - | 12032 | 0 | 0 | - |
| S2 | Lijian Base of GAAS | 404.19 | - | NoCt | - | 11563 | 0 | 0 | - |
| S3 | Lijian Base of GAAS | 218.24 | - | NoCt | - | 9433 | 0 | 0 | - |
| S4 | Lijian Base of GAAS | 262.25 | - | NoCt | - | 14578 | 0 | 0 | - |
| S5 | Lijian Base of GAAS | 322.9 | - | 40.71 | - | 11521 | 0 | 0 | - |
| S6 | Lijian Base of GAAS | 307.05 | - | NoCt | - | 13348 | 0 | 0 | - |
| S7 | Lijian Base of GAAS | 359.13 | - | 42.48 | - | 10773 | 0 | 0 | - |
| S8 | Lijian Base of GAAS | 321.28 | - | NoCt | - | 9970 | 0 | 0 | - |
| S9 | Lijian Base of GAAS | 359.74 | - | NoCt | - | 13272 | 0 | 0 | - |
| S10 | Lijian Base of GAAS | 330.38 | - | NoCt | - | 7820 | 0 | 0 | - |
| S11 | Lijian Base of GAAS | 239.94 | - | NoCt | - | 7100 | 0 | 0 | - |
| S12 | Lijian Base of GAAS | 299.04 | - | NoCt | - | 7345 | 0 | 0 | - |
| S13 | Longtou Township, Fusui County, Chongzuo City | 611.8 | - | 45 | - | 65316 | 0 | 0 | - |
| S14 | Longtou Township, Fusui County, Chongzuo City | 3876.8 | + | 36.97 | + | 63461 | 71 | 0.1118797 | + |
| S15 | Longtou Township, Fusui County, Chongzuo City | 3760.7 | + | 37.62 | + | 58462 | 20 | 0.0342103 | + |
| S16 | Longtou Township, Fusui County, Chongzuo City | 919.5 | + | 41.91 | - | 56248 | 5 | 0.0088892 | + |
| S17 | Longtou Township, Fusui County, Chongzuo City | 5006.2 | + | 32.16 | + | 64089 | 601 | 0.9377584 | + |
| S18 | Longtou Township, Fusui County, Chongzuo City | 4317.3 | + | 34.74 | + | 48601 | 79 | 0.1625481 | + |
| S19 | Longtou Township, Fusui County, Chongzuo City | 3418.9 | + | 39.34 | + | 72712 | 156 | 0.2145451 | + |
| S20 | Longtou Township, Fusui County, Chongzuo City | 1352 | + | 37.66 | + | 62419 | 9 | 0.0144187 | ＋ |

Note: ΔRn, normalized reporter fluorescence signal; Ct, cycle threshold; NoCt, no detectable amplification (i.e., negative result). (+) and (-) indicate positive and negative detection results, respectively. Relative abundance was calculated as the ratio of S. rolfsii reads to total reads (%).

References

Feng, H., Chen, J., Yu, Z., Li, K., Li, Z., and Li, Y., et al. (2020). Pathogenicity and fungicide sensitivity of Pythium and Phytopythium spp. associated with soybean in the Huang-Huai region of China. Plant Pathol. 69, 1083-1092. doi: 10.1111/ppa.13187
